# Deepure Tea Improves High Fat Diet-Induced Insulin Resistance and Nonalcoholic Fatty Liver Disease

**DOI:** 10.1155/2015/980345

**Published:** 2015-10-04

**Authors:** Jing-Na Deng, Juan Li, Hong-Na Mu, Yu-Ying Liu, Ming-Xia Wang, Chun-Shui Pan, Jing-Yu Fan, Fei Ye, Jing-Yan Han

**Affiliations:** ^1^Tasly Microcirculation Research Center, Peking University Health Science Center, Beijing 100191, China; ^2^Key Laboratory of Microcirculation, State Administration of Traditional Chinese Medicine of the People's Republic of China, Beijing 100191, China; ^3^Key Laboratory of Stasis and Phlegm, State Administration of Traditional Chinese Medicine of the People's Republic of China, Beijing 100191, China; ^4^Institute of Materia Medica, Chinese Academy of Medical Science and Peking Union Medical College, Beijing 100050, China; ^5^Department of Integration of Chinese and Western Medicine, School of Basic Medical Sciences, Peking University, Beijing 100191, China

## Abstract

This study was to explore the protective effects of Deepure tea against insulin resistance and hepatic steatosis and elucidate the potential underlying molecular mechanisms. C57BL/6 mice were fed with a high fat diet (HFD) for 8 weeks to induce the metabolic syndrome. In the Deepure tea group, HFD mice were administrated with Deepure tea at 160 mg/kg/day by gavage for 14 days. The mice in HFD group received water in the same way over the same period. The age-matched C57BL/6 mice fed with standard chow were used as normal control. Compared to the mice in HFD group, mice that received Deepure tea showed significantly reduced plasma insulin and improved insulin sensitivity. Deepure tea increased the expression of insulin receptor substrate 2 (IRS-2), which plays an important role in hepatic insulin signaling pathway. Deepure tea also led to a decrease in hepatic fatty acid synthesis and lipid accumulation, which were mediated by the downregulation of sterol regulatory element binding protein 1c (SREBP-1c), fatty acid synthesis (FAS), and acetyl-CoA carboxylase (ACC) proteins that are involved in liver lipogenesis. These results suggest that Deepure tea may be effective for protecting against insulin resistance and hepatic steatosis via modulating IRS-2 and downstream signaling SREBP-1c, FAS, and ACC.

## 1. Introduction

Nonalcoholic fatty liver disease (NAFLD) and insulin resistance are the main pathophysiological characteristic of metabolic syndrome [1], which has become a significant public health problem as a result of high fat diet and sedentary lifestyles [2, 3]. NAFLD, once considered benign, may progress to steatohepatitis, fibrosis, and ultimately cirrhosis [4–6]. NAFLD represents a state of lipid accumulation in hepatocytes, and its pathogenesis is associated with enhanced liver lipogenesis and hepatic insulin resistance. The liver lipogenesis can be activated by elevated plasma insulin, as seen in patients with the metabolic syndrome, type 2 diabetes, or obese individuals [4, 7].

In the liver, insulin is involved in a number of actions responsible for glucose control and lipid metabolism. Insulin receptor substrate (IRS) proteins are a family of cytoplasmic adaptor proteins that transmit signal from insulin receptor to its final biological actions through a series of intermediate effectors. Hepatic insulin signaling for these effects is mediated mainly through insulin receptor substrate 2 (IRS-2) [8, 9], rather than insulin receptor substrate 1 (IRS-1). Elevated insulin also leads to activation of the lipid biosynthetic pathway through activation of the expression and proteolytic maturation of the transcription factor sterol regulatory element binding protein 1c (SREBP-1c) [10], thereby leading to the increased expression of fatty acid synthase (FAS) and acetyl-CoA carboxylase (ACC) in the lipogenesis pathway, resulting in steatosis. Moreover, NAFLD leads to hepatic insulin resistance by stimulating gluconeogenesis [11], and upregulated SREBP-1c may suppress IRS-2-mediated insulin signaling generating a feedforward machinery to further stimulate or worsen NAFLD [11, 12]. Thus, NAFLD and insulin resistance have a number of reciprocal relationships and can enhance each other [13, 14].

Tea is one of the most popular beverages worldwide and can be categorized into three types: nonfermented green, partially fermented oolong, and fully fermented black and Pu-erh tea [15]. Several biological functions of Pu-erh tea have been reported, such as antiobesity [16], antihyperlipidemia [17], anti-liver fat accumulation [18], and promoting skeletal muscle glucose transport [19]. Also, epigallocatechin gallate, a compound from tea, has been shown to reduce intestinal lipid absorption [20] and lower blood lipids [21]. Therefore, the mechanisms of Pu-erh tea protecting against obesity-associated disease are likely to be multifaceted. However, the study is limited so far to address the mechanism underlying the actions of Pu-erh tea.

The present study was conducted to investigate whether Deepure tea, a specific tea concentrated from Pu-erh tea, could ameliorate insulin resistance and NAFLD in high fat diet (HFD) raised mice and possible mechanisms of its action. We demonstrated that Deepure tea decreased HFD-induced hyperinsulinemia and improved diet-induced NAFLD in C57BL/6 mice. HFD markedly inhibited hepatic IRS-2 protein expression in mice, which was reversed by treatment with Deepure tea. The improved hepatic steatosis appears to be mediated through the downregulation of SREBP-1c protein level, subsequently decreasing the level of FAS and ACC, which are involved in de novo lipogenesis in the liver.

## 2. Materials and Methods

### 2.1. Materials

Deepure tea was supplied by Tasly Pharmaceutical Co. Ltd. (Tianjin, China). The raw materials were extracted from leaves of old Pu-erh tea trees, which were from Yunnan province of China. The batch number of the Deepure tea used in this experiment was 20110918. The processing of the product followed a strict quality control, and the ingredients were subjected to standardization. Deepure tea was manufactured as nanometer level powder after dynamic cycle extraction and concentrated by evaporating and spray drying.

Antibodies recognizing IRS-2 and GAPDH were from Cell Signaling Technology (Boston, MA, USA). Antibodies against SREBP-1c, FAS, and ACC were from Abcam (Cambridge, MA, USA). BCA protein assay kit was purchased from Applygen Technologies (Beijing, China). ELISA kit for LDLR of mice was purchased from Andygene (Richardson, USA). All other reagents used in our study were of analytical grade.

### 2.2. Animal Model and Treatment

Four-week-old male C57BL/6 mice (the animal certificate number was SCXK (Jing) 2006–2009) were purchased from Weitonglihua Animal Center, Beijing, China. The animals were housed at 21–23°C and a humidity level of 40–60%. They were exposed to a 12 h lighting cycle and allowed* ad libitum* access to water and the appointed chows. The HFD-induced mice were fed with HFD for 8 weeks, which contained 50% fat (mainly from lard), 36% carbohydrate, and 14% protein, with a total energy content of 21.0 kJ/g. In control group, aged-matched male C57BL/6 mice were fed with standard laboratory chows containing 12% fat, 62% carbohydrate, and 26% protein [22] with a total energy content of 12.6 kJ/g. The HFD-induced mice were randomly divided into two groups: HFD and Deepure tea. The animals in Deepure tea group received Deepure tea (160 mg/kg/day) orally for 14 days. The mice in HFD and control groups were given the same volume of water. All animal experiments were approved by the Beijing Municipal Ethics Committee for Laboratory Animals.

### 2.3. Intraperitoneal Glucose Tolerance Test (IPGTT)

C57BL/6 mice were fasted for 2 h before experiment. Blood samples were collected from tail veins for determination of baseline values of blood glucose (*t* = 0 min). The mice were then injected intraperitoneally with glucose at 2 g/kg, and additional blood samples were collected at 15, 60, and 120 min, respectively, for glucose measurement.

### 2.4. Biochemical Assays

After C57BL/6 mice were fasted for 6 h, tail vein blood was collected. The samples were centrifuged at 3500 rpm for 10 min at 4°C to separate plasma. Plasma insulin levels were measured by enzyme-linked immunosorbent assay (ELISA) using an insulin ultrasensitive ELISA kit (ALPCO, Salem, NH, USA), according to the manufacturer's instruction. The concentrations of plasma triglyceride (TG) and total cholesterol (TC) were determined according to the kit instruction (Jian Cheng Biotechnology Company, Nanjing, China). Plasma glucose levels were detected to calculate the homeostasis model assessment (HOMA). HOMA-IR index = fasting blood glucose (mmol/L) × fasting plasma insulin (pmol/L)/22.5 [23]. The concentration of LDLR protein in liver tissues was assessed by ELISA kit according to the manufacturer's protocol. OD values were determined by enzyme microplate reader (Thermo Multiskan Mk3, Thermo Fisher Scientific Inc., Barrington, USA), with detection wave length of 450 nm. The LDLR level was calculated based on the standard curves.

### 2.5. Western Blotting

Liver tissues were lysed in sample buffer containing 62 mM Tris-HCl, pH 6.8, 0.1% SDS, 0.1 mM sodium orthovanadate, and 50 mM sodium fluoride. The protein content was determined by the BCA protein assay. Equal amounts of proteins were loaded and separated by SDS-PAGE. After electrophoresis, the proteins were transferred on membranes, after being blocked with 3% nonfat dry milk the membrane with target proteins was incubated with an antibody against IRS-2, SREBP-1c, ACC, FAS, or GAPDH overnight at 4°C. The blots were incubated with a respective HRP-conjugated second antibody, and then immunoreactive bands were revealed using an enhanced chemiluminescence system (Applygen Technologies Inc., Beijing, China). The protein signal was quantified by scanning densitometry in the X-film by Image-Pro Plus 6.0 software (Bio-Rad, Hercules, California, USA) [24].

### 2.6. Hematoxylin and Eosin (HE) Staining

Liver tissue samples from each mouse were fixed in formalin saline solution (10%) and then embedded in paraffin, sliced at five micrometer thickness, and stained with HE for histological analysis under a light microscope.

### 2.7. Statistical Analysis

Data were expressed as means ± SEM. Student's *t*-test for unpaired observations was used to compare the mean values of two groups. A value of *p* < 0.05 was considered statistically significant.

## 3. Results

### 3.1. Deepure Tea Treatment Improves Hyperinsulinemia and Insulin Resistance in HFD Mice

**Figure 1:**
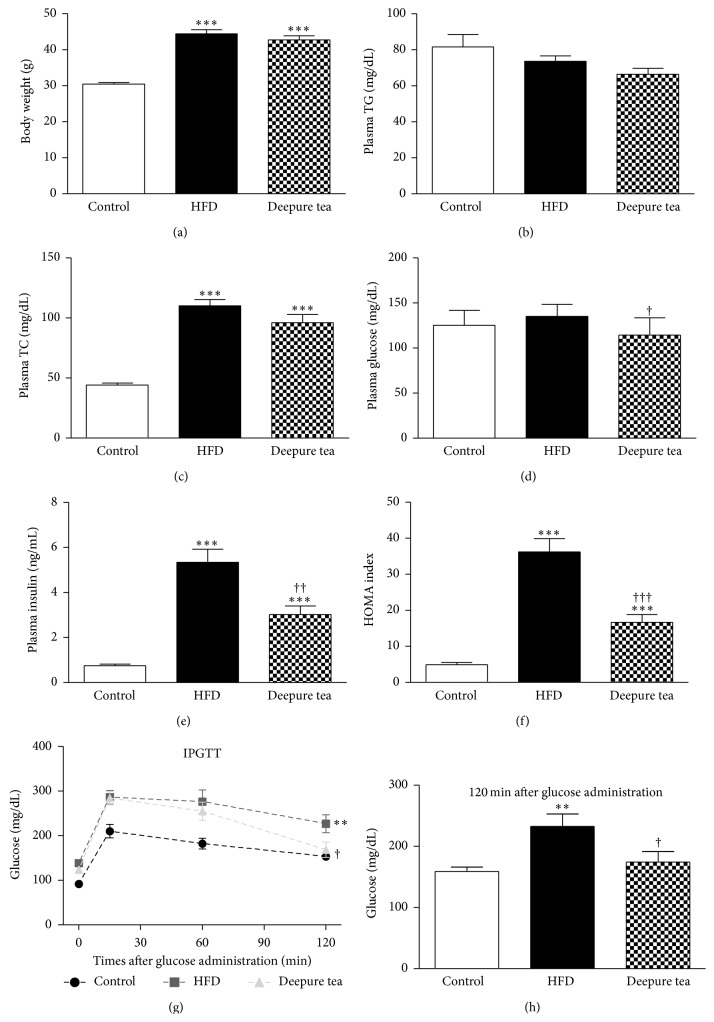
Deepure tea restored plasma level of insulin and improved insulin resistance in high fat diet mice. Plasma parameters were measured in the fasting state of the mice. (a) The body weight in different groups. (b)-(c) Plasma TG and TC level in different groups. (d) Plasma glucose level in different groups at baseline. (e) Plasma insulin level in different groups at baseline. (f) Insulin resistance of mice evaluated by homeostasis model assessment (HOMA) index. (g) Intraperitoneal glucose tolerance test (IPGTT). (h) Plasma glucose concentration tested 120 min after glucose administration. The number of animals included was 10 (control group), 9 (HFD group), and 8 (Deepure tea group), respectively. All experiments were performed in triplicate. Data are means ± SEM. ^*∗*^ *p* < 0.05, ^*∗∗*^ *p* < 0.01, and ^*∗∗∗*^ *p* < 0.001 versus control mice; ^†^ *p* < 0.05 and ^††^ *p* < 0.01 and ^†††^ *p* < 0.001 versus HFD mice.

The mice in HFD and Deepure tea groups did not show any difference in body weight but both possessed a higher body weight than those in the control group, as shown in Figure 1(a). After 6-hour fasting, the HFD mice demonstrated hypercholesterolemia, but not hypertriglyceridemia, as compared with the normal control mice (Figures 1(b) and 1(c)). Plasma TC was slightly decreased in Deepure tea groups compared to the HFD group (93.13 ± 6.799 versus 106.9 ± 5.229), but without significance.

At baseline, plasma glucose level in HFD group did not differ from that in control group but decreased by 15.3% in Deepure tea treated group with significance (Figure 1(d)). In contrast, HFD resulted in a significant increase in serum insulin, as compared with the control group, which was reduced as much as 43.5% by Deepure tea treatment (Figure 1(e)). The homeostasis model assessment (HOMA) was calculated as an index of insulin resistance. As shown in Figure 1(f), HOMA-IR index was decreased by ~54% in Deepure tea treated mice compared with HFD mice. The potential of Deepure tea treatment to improve insulin resistance was also confirmed by IPGTT (Figure 1(g)).  Figure 1(h) presents the results of the plasma glucose levels detected 2 hours after glucose administration, showing a decrease by 25.1% in Deepure tea-treatment mice compared with HFD mice.

### 3.2. Deepure Tea Attenuates HFD-Induced Hepatic Steatosis in C57BL/6 Mice

**Figure 2:**
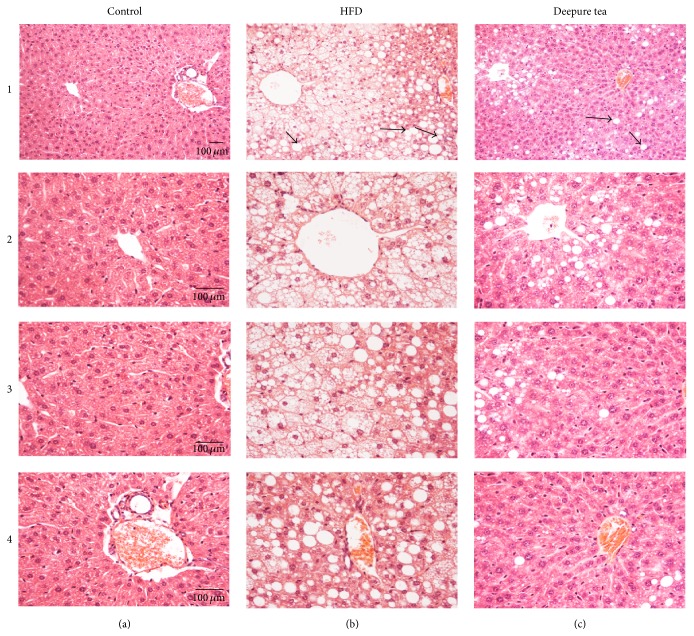
Deepure tea diminished nonalcoholic steatohepatitis caused by HFD in C57BL/6 mice. Representative images of liver tissues collected from the mice of control (a), HFD (b), and Deepure tea (c) group with low magnification displayed in 1 and high magnification in 2, 3, and 4 are shown. The number of mice examined in each group was 6. Bar = 100 *μ*m.

Histological examination was conducted at the end of the experiment, and the result is illustrated in Figure 2. Strikingly, HFD led to an apparent NAFLD compared with control mice, which manifested a large number of lipid-filled vacuoles (arrowheads) in liver tissue. Impressively, liver sections from Deepure tea-treatment mice revealed an obvious reduction of lipid droplets (arrowheads), showing the potential of Deepure tea to relieve hepatic steatosis.

### 3.3. Deepure Tea Modulates the Expression of Genes Involved in Hepatic Insulin Signaling Pathway and Hepatic Lipid Synthesis

**Figure 3:**
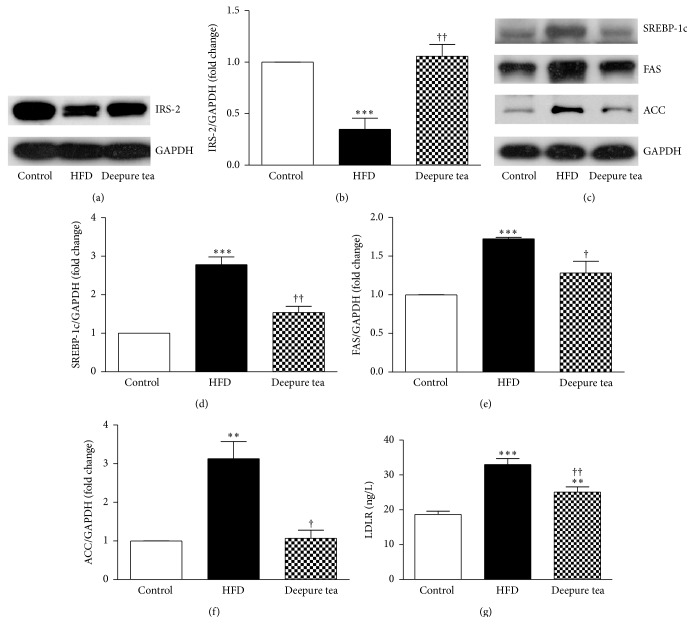
Deepure tea modulated the expression of genes involved in hepatic insulin signaling and lipid metabolism. (a) Representative western blot of IRS-2 expression in liver tissue of mice in different group. (b) Quantification of the western blot results of IRS-2. (c) Representative western blot of SREBP-1c, FAS, and ACC expression in liver tissue of mice in different group. (d) Quantification of the western blot results of SREBP-1c. (e) Quantification of the western blot results of FAS. (f) Quantification of the western blot results of ACC, *N* = 6. (g) LDLR levels determined by ELISA kit, *N* = 8. The results are presented as mean ± SEM of 3 experiments. ^*∗∗*^ *p* < 0.01 and ^*∗∗∗*^ *p* < 0.001 versus control mice; ^†^ *p* < 0.05 and ^††^ *p* < 0.01 versus HFD mice.

Insulin resistance is known showing a reduced insulin sensitivity of peripheral tissue with aberrant IRS-2 and downstream members of the insulin signaling pathway [25]. To examine whether IRS-2 expression is changed in the present setting, we assessed the IRS-2 protein in liver tissues. As an important regulator of liver insulin signaling, the expression of IRS-2 was markedly reduced in the mouse liver of the HFD group, as compared to those in normal diet mice. Significantly, mice that received Deepure tea showed evidently higher IRS-2 protein level in comparison with that in HFD mice (Figures 3(a) and 3(b)).

To gain insight into the protective mechanisms of Deepure tea against insulin resistance and hepatic steatosis in HFD-fed mice, we further examined the protein levels in hepatic tissue that are involved in insulin signaling and lipogenesis. SREBP-1c is a key transcriptional factor regulating de novo lipogenesis in liver [26]. As shown in Figures 3(c) and 3(d), HFD markedly upregulated hepatic SREBP-1c expression. Of notice, hepatic SREBP-1c protein level of the Deepure tea group was reduced by 44.7%, compared to the HFD group. Similar results were observed for the protein levels of hepatic FAS and ACC, the target gene of SREBP-1c and the key enzyme of de novo lipogenesis, which were downregulated by 25.6% (Figure 3(e)) and 65.8% (Figure 3(f)), respectively, in the Deepure tea-treatment mice, compared to the HFD group. Low density lipoprotein receptor (LDLR) plays an important role in insulin resistance [27, 28]. The level of LDLR was detected by ELISA kit. As shown in Figure 3(g), LDLR was significantly upregulated in liver tissues from HFD mice. After treatment with 160 mg/kg/day Deepure tea, the upregulation of liver LDLR induced by HFD was reduced.

## 4. Discussion

This study provides evidence that short-term intake of Deepure tea protects against the development of hyperinsulinemia and NAFLD in HFD mice. Histologic results clearly showed that 14-day feeding of Deepure tea at 160 mg/kg reduced dietary-induced hepatic steatosis, which was correlated with the downregulation of SREBP-1c, FAS, and ACC expression in the liver. Consistent with reduced plasma insulin, the hepatic IRS-2 expression was significantly reduced in HFD mice. Thus, the findings from our investigation suggest that Deepure tea may be a desirable food for preventing insulin resistance and ectopic lipid accumulation, especially in HFD-induced obesity.

High-energy diets are used widely in nutritional experiments as a strategy to induce obesity in animals [29]. Rodents fed a lard-based HFD are reported to exhibit visceral adiposity, dyslipidemia, hyperinsulinemia, and NAFLD [22], which are typically linked with human obesity. In line with this report, mice in the preset study were fed with high fat diet containing 50% fat, 36% carbohydrate, and 14% protein for 8 weeks and developed obesity, insulin resistance, and lipid metabolic disorder. Insulin resistance is one of the key pathogenic factors of the metabolic syndrome [30]. Impaired glucose tolerance assessed by IPGTT indicates the presence of insulin resistance and abnormality in glucose disposal [31]. The present study demonstrated that Deepure tea effectively decreased plasma insulin and ameliorated glucose tolerance in the high fat diet mice. A similar phenomenon has also been reported for the water extract of Pu-erh tea showing its ability to inhibit the increase in blood insulin and improve impaired glucose tolerance in db/db mice [32]. Also, we found that administration of Deepure tea for 14 days at 160 mg tea/kg body weight, a dose that is approximately equivalent to 1 g per day recommended for human, significantly repressed the elevated HOMA-IR index in HFD mice. Taken together, Deepure tea can improve insulin resistance induced by a high fat diet in C57BL/6 mice. The amount of food intake was almost the same for the mice in HFD and Deepure tea group (data not shown), and the body weight of mice did not reveal significant difference between HFD group and Deepure tea group. Plasma TC was only slightly decreased in Deepure tea group mice compared to that of the HFD group, which may result from the relatively short-term administration of Deepure tea.

Insulin resistance manifests reduced insulin sensitivity of peripheral tissue with an abnormality in the insulin signaling pathway, including IRS and other downstream molecules [33]. IRS-2 is the main mediator of hepatic insulin signaling, controlling hepatic insulin sensitivity [9]. In the present study, there was a dramatic decrease in hepatic IRS-2 in the liver of HFD mice. Deepure tea intragastrically given for 14 days could reverse the downregulation of IRS-2 protein in dietary-induced obese mice, which may be contributable to the improvement of hepatic insulin resistance. This result is in line with previous study showing that the water extract of Pu-erh tea can significantly increase glucose uptake by HepG2 cell [32]. Nonetheless, the present study is the first to show a possible molecular mechanism for Pu-erh tea to improve hepatic insulin resistance. However, more researches are needed to elucidate the mechanism that Deepure tea ameliorates dietary-induced hepatic insulin resistance.

Excessive intake of fatty acid will lead to fatty liver. In the present study, C57BL/6 mice fed with high fat diet displayed NAFLD, whereas after administration of Deepure tea, the lipid content in liver was significantly reduced, suggesting that the Deepure tea could significantly improve hepatic lipid accumulation.

The increased fatty acid de novo synthesis is one of the major sources of lipid accumulation in liver. The protein SREBP-1c, as a transcriptional regulator of lipid synthesis, activates genes required for de novo lipogenesis [26, 34–36]. SREBP-1c activates ACC that produces malonyl-CoA at the mitochondrial membrane and also increases the expression of FAS, which plays an important role in the lipogenesis pathway. Previously, Pu-erh tea [18], Yerba Mate tea [37], and coffee polyphenols [38] have been shown to decrease the hepatic SREBP-1c mRNA level, resulting in the suppression of body fat accumulation. Here, we observed that the proteins of SREBP-1c, FAS, and ACC were increased impressively in the liver of high fat diet mice, which were significantly restored by treatment with Deepure tea, accompanied with an attenuation of the lipid accumulation. Deepure tea also reduced the upregulated LDLR protein induced by high fat diet. However, further investigations are required to clarify the effects of Deepure tea on the expressions of genes involved in triglyceride metabolism such as fatty acid oxidation, lipolysis, and lipid delivery.

In conclusion, this study demonstrates that short-term oral administration of Deepure tea has beneficial effects on diet-induced hyperinsulinemia and ectopic lipid accumulation. These effects are most likely mediated through modification of IRS-2 and its downstream signaling SREBP-1c, FAS, and ACC. Importantly, the dose of Deepure tea administrated in the present study was close to that which human intakes as routing. Thus, these results also suggest that regular Deepure tea consumption is conducive to maintain metabolism homeostasis.
